# Comparative evaluation of the shaping performance of three NiTi rotary systems in distolingual canals of 3D-printed three-rooted mandibular first molars

**DOI:** 10.3389/fdmed.2026.1742809

**Published:** 2026-01-27

**Authors:** Bingbing Bai, Panpan Zhang, Tao Yang, Fan Pei, Nan Geng, Yongchun Gu

**Affiliations:** 1 General Dentistry Department, The Stomatology Hospital Affiliated of Suzhou Vocational Health College, Suzhou, China; 2 Department of Dentistry and Central Laboratory, Ninth People’s Hospital of Suzhou, Soochow University, Suzhou, China; 3 State Key Laboratory Cultivation Base of Research, Prevention and Treatment for Oral Diseases, Jiangsu Province Engineering Research Center of Stomatological Translational Medicine, Department of VIP Clinic, Nanjing Medical University, Affiliated Stomatological Hospital of Nanjing Medical University, Nanjing, China

**Keywords:** distolingual canal, nickel-titanium endodontic instrument, root canal instrumentation, three-dimensional printing, three-rooted mandibular first molar

## Abstract

**Introduction:**

This *ex vivo* study compared the shaping ability of three nickel-titanium (NiTi) rotary systems in distolingual (DL) canals using 3D-printed three-rooted mandibular first molar (3RM1) replicas.

**Materials and methods:**

Two extracted 3RM1s with DL roots (curvatures: 21.9 ° and 37.5 °) were selected and replicated via 3D printing. Eighteen resin replicas per specimen were equally divided into three groups for instrumentation with OneShape (OS), WaveOne Gold (WOG), or ProTaper Universal (PTU), following manufacturers' protocols. Micro-computed tomography (micro-CT) scans were performed before and after DL canal instrumentation. Canal straightening, volume/surface area changes, maximum cutting thickness (MCT), and residual wall thickness (RWT) at cervical and furcation regions were analyzed.

**Results:**

PTU cause the most canal straightening (Specimen 1 replicas: 5.4 ± 0.4 °; Specimen 2 replicas: 11.0 ± 1.5 °), largest increases in canal volume (Specimen 1 replicas: 106.9 ± 41.0%; Specimen 2 replicas: 174.6 ± 26.8%) and surface area (Specimen 1 replicas: 40.3 ± 17.5%; Specimen 2 replicas: 60.0 ± 16.7%), thinnest RWT and highest MCT (Specimen 1 replicas: 0.28 ± 0.04 mm; Specimen 2 replicas: 0.39 ± 0.04 mm). Conversely, WOG showed minimal canal straightening (Specimen 1 replicas: 2.5 ± 0.40 °; Specimen 2 replicas: 4.8 ± 1.5 °), the least canal enlargement (Volume: 30.8 ± 13.1% and 78.6 ± 35.3%; Area: 10.1 ± 8.8% and 23.6 ± 15.3%), greatest RWT, and lowest MCT (Specimen 1 replicas: 0.20 ± 0.04 mm; Specimen 2 replicas: 0.30 ± 0.07 mm). OS performed intermediately. MCT consistently occurred at 2−3 mm below the furcation.

**Conclusion:**

Both single-file systems (WOG and OS) effectively shaped DL canals while maintaining proper geometry. However, OS requires caution in severe curvatures. PTU's aggressive cutting caused excessive resin removal, limiting its DL canal suitability.

## 1 Introduction

The permanent mandibular first molar typically possesses two roots and three canals (two mesial and one distal canal). However, in certain cases, a third root may be identified on the distolingual (DL) aspect of the tooth (1, 2). This root variation is influenced by ethnicity, with a high prevalence rate ranging from 5% to 40% in Mongolia populations, whereas the frequency is generally below 5% in Caucasian populations and less than 3% in African populations (3–5). In endodontic practice, the presence of a DL root/canal poses clinical challenges (2). Conventional periapical radiographs often fail to detect the DL root due to anatomical superimposition of distal roots and surrounding alveolar bone, leading to missed diagnosis (6). This radiographic limitation, combined with the DL root's characteristic morphological variability (typically smaller diameter and distinct buccal curvature) (2, 7), creates technical difficulties during instrumentation. Such anatomical complexity predisposes to iatrogenic errors including ledging, zipping, apical/strip perforations, and instrument separation (8).

**Figure 1:**
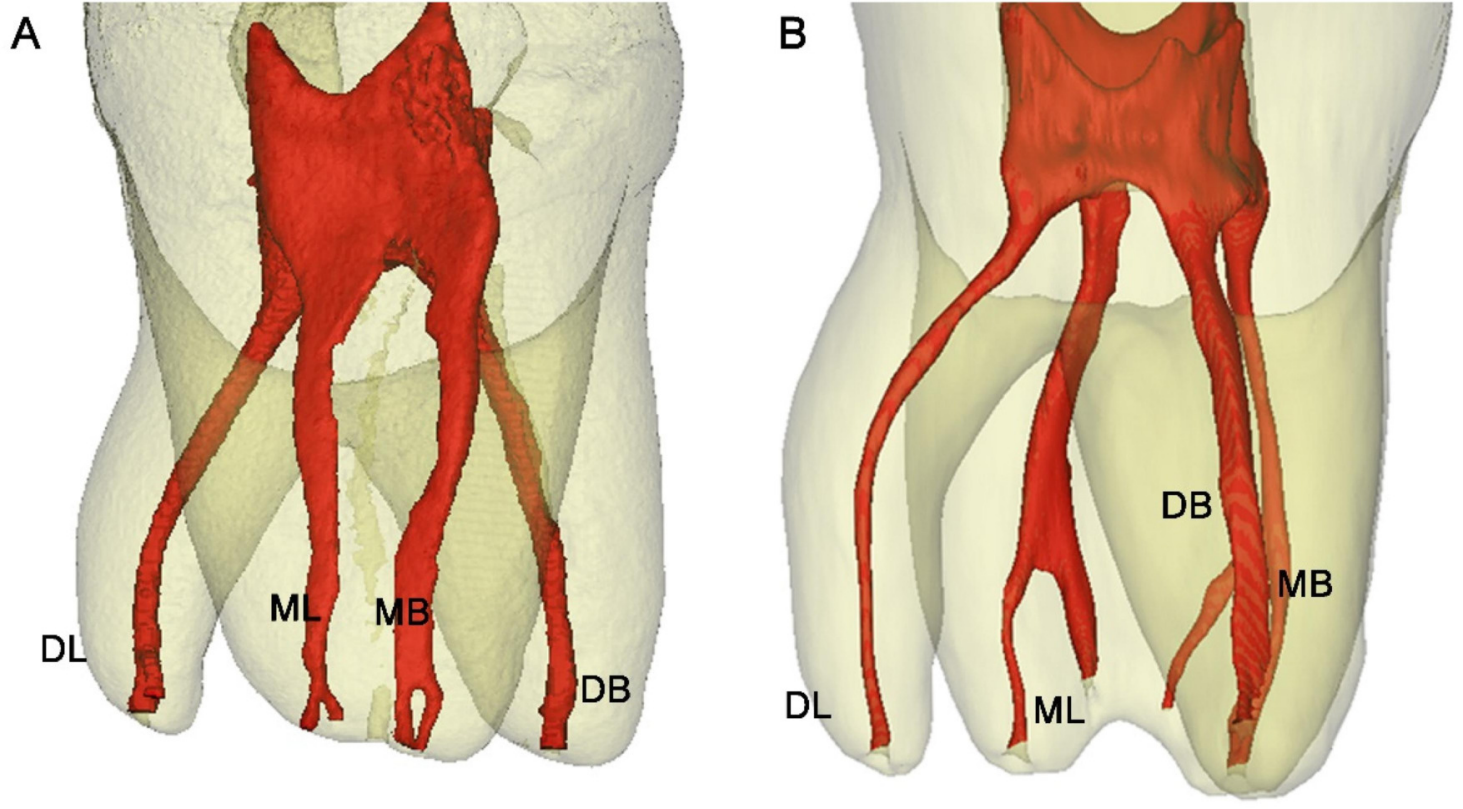
Micro-CT 3D reconstructed images of two representative tooth specimens selected as prototypes for 3D printing. **(A)** Specimen 1: A three-rooted mandibular first molar with a distolingual (DL) canal curvature of 21.9 °; **(B)** Specimen 2: A three-rooted mandibular first molar with a DL canal curvature of 37.8 °. DL, DB, ML and MB denote the distolingual, distobucal, mesiolingual and mesiobucal canals, respectively.

Over the past two decades, the endodontic field has witnessed the introduction of numerous nickel-titanium (NiTi) file systems, many of which have gained widespread clinical adoption (9–11) Manufacturers promote these systems as offering enhanced shaping efficiency and reduced risk of iatrogenic complications, attributing these advantages to their advanced design features, improved alloy compositions, and optimized motion kinematics (10, 11). However, substantial clinical evidence underscores that root canal anatomy remains the most critical factor influencing treatment outcomes. This reality necessitates that clinicians possess an in-depth understanding of both the operational characteristics and limitations of their selected NiTi system. Prudent instrument selection should be guided by comprehensive preoperative evaluation of root canal morphology, coupled with careful consideration of the specific instrument's design parameters, to ensure optimal procedural safety and effectiveness. The use of fewer instruments can simplify root canal preparation. Single-file NiTi systems thus provide distinct advantages, including reduced instrumentation time and minimized cross-contamination risk (12). The OneShape (OS; MicroMega, Besançon, France) system utilizes a conventional austenitic 55-NiTi alloy and consists of a single file (size 25/6%) with a passive tip and a variable cross-sectional design. This design includes a triangular cross-section with three sharp cutting edges in the apical and middle portions, which progressively transition to two cutting edges near the shaft (12). In contrast, the WaveOne Gold (WOG; Dentsply Maillefer, Ballaigues, Switzerland) system retains the reciprocating motion of its predecessor (WaveOne) but incorporates modified dimensions and geometry (13). The WOG system has an off-center parallelogram cross-section with two cutting edges and consists of four instruments: 21/6% (small), 25/7% (primary), 35/6% (medium) and 45/5% (large). This differs from the original WaveOne system, which employed a convex triangular cross-section with larger tapers and dimensions (14, 15). Additionally, WOG files undergo a proprietary gold heat treatment—a thermal process involving controlled heating and slow cooling—to enhance their flexibility and cyclic fatigue resistance. Kinematically, the WOG system operates with a 170 ° counterclockwise (cutting) motion followed by a 50 ° clockwise (release) motion, incorporating a reverse helix design and a semi-active guiding tip (16, 17). ProTaper Universal (PTU; Dentsply Maillefer, Switzerland) represents one of the most widely used rotary sequential instrumentation systems, characterized by its convex triangular cross-sectional design and progressive taper configuration. The shaping performance of PTU instruments has been frequently employed as a reference standard when evaluating newer generation NiTi systems (18, 19). To our knowledge, this study represents the first systematic comparison of these three NiTi rotary systems in the challenging anatomical context of DL canals in three-rooted mandibular first molars (3RM1s).

Three-dimensional (3D) printing technology has emerged as a valuable tool in endodontic research, enabling rapid and precise fabrication of anatomical replicas (20, 21). This innovative approach involves reconstructing digital tooth models from micro-computed tomography (micro-CT) or cone-beam CT (CBCT) scans of natural teeth, which are then exported in standard tessellation language (STL) format for 3D printing. The resulting replicas, frequently produced using advanced photo-polymerizable resins, provide numerous advantages over extracted teeth specimens, including controlled anatomical parameters, elimination of cross-infection risks, circumvention of ethical procurement challenges, and establishment of repeatable experimental conditions (22–25). Within this methodological framework, the present micro-CT study was designed to conduct a comparative evaluation of three contemporary NiTi instrumentation systems (OS, WOG, and PTU) specifically focusing on their shaping efficacy in the DL canals of 3RM1 replicas, thereby addressing critical gaps in our understanding of instrument performance in complex root canal anatomies. The null hypothesis was that there would be no significant differences among the three NiTi systems in shaping ability when preparing DL canals in standardized 3D-printed replicas of 3RM1s.

## 2 Materials and methods

### 2.1 Acquisition of micro-CT imaging data from extracted teeth

Micro-CT imaging data from 20 extracted 3RM1s were acquired from a prior study [2]. All specimens were obtained from ethnically Chinese patients whose teeth required extraction due to periodontal disease, non-restorable carious lesions, or prosthodontic indications. Specimens meeting any of the following exclusion criteria were omitted from the study: (a) teeth with incomplete root formation, (b) previously endodontically treated teeth, and (c) teeth exhibiting significant structural defects or root fractures.

Each tooth was scanned along its axis using micro-CT scanning (SkyScan1174; Bruker-microCT, Kontich, Belgium) at a voxel size of 21.6 μm, with 800 μA, a rotation step of 0.7°, 50 kVp, one frame averaging, and an arch rotation of 180°. The acquired micro-CT datasets were subsequently imported into Mimics 21.0 (Materialise, Leuven, Belgium) for 3D reconstruction of both dental anatomy and root canal systems.

### 2.2 3D printing of the 3RM1s

A comprehensive analysis of root canal morphology was performed, with particular emphasis on the curvature characteristics of the DL canal/root. The overall mean curvature of DL canals across all 20 specimens was 32.1 ° (range: 14–57 °) (2), as determined by Schneider's method (26) in the proximal view. For 3D prototyping, two representative specimens (Figure 1) were selected from the lower quartile (Specimen 1, 21.9 °) and the upper quartile (Specimen 2, 37.5 °) of the curvature distribution, thereby providing representative examples of less complex (moderate) and more complex (severe) anatomical challenges, without being the absolute minima or maxima. Notably, both DL canal curvatures were predominantly aligned along the sagittal plane. Additional geometric parameters of the DL canals are detailed in Table 1.

**Table 1:** Geometric parameters of the DL canals of the two prototype specimens.

Geometric parameter	Specimen 1	Specimen 2
Canal length (mm)	8.39	9.95
Root length (mm)	6.98	7.22
*D* of apical foramen (μm)	194	190
*d* of apical foramen (μm)	155	151
Canal curvature ( °)	21.9	37.5

*D* maximum diameter; *d* minimum diameter.

The tooth replicas were fabricated using a 3D printing protocol as previously described (25). To ensure experimental consistency and minimize coronal preparation-related variability, digital sectioning was performed 2 mm coronal to the cementoenamel junction (CEJ) using Mimics software, fully exposing the pulp chamber floor and root canal orifices. The processed 3D models were exported as STL files and printed using a high-precision resin-based 3D printer (Saturn 2, ELEGOO, China) with spatial resolutions of 28.5 μm in the XY plane and 20 μm along the Z-axis. MOLEGRID™ Ultradetail photopolymer resin (KEXCELLED, Suzhou, China) served as the printing material. According to the manufacturer's specifications, this rigid photopolymer resin exhibits a Shore D hardness of 84 following standard LCD polymerization. This process yielded 36 identical tooth replicas (*n* = 18 per prototype specimen).

### 2.3 Root canal instrumentation

The 18 resin replicas derived from each specimen were randomly divided into three groups (*n* = 6 per group) for DL canal preparation using three different NiTi systems, in strict accordance with the manufacturers' recommended protocols. All instrumentation procedures were performed by a single operator (B.B.), a general dentist with three years of clinical experience, to eliminate inter-operator variability. Prior to the study, the operator underwent specialized hands-on training for each NiTi system, utilizing both extracted natural teeth and resin replicas, under the direct supervision of an experienced endodontist (Y.G.) with over 27 years of clinical practice. Training proceeded until consistent and reproducible proficiency was demonstrated, strictly following the manufacturers' guidelines.

Following confirmation of apical patency using #8 and #10 K-files (Dentsply Sirona, Ballaigues, Switzerland), a glide path preparation was established by sequential use of #10 and #15 K-files to the predetermined working length (1 mm short of the apical foramen). All NiTi instrumentation was performed using an X-Smart Plus endodontic motor (Dentsply Maillefer, Switzerland) with strict adherence to single-use protocols for all files. Each single-file instrument was employed in a slow in-and-out pecking motion with an amplitude of approximately 3 mm. The debris on the files was cleaned with wet gauze, and the canal was irrigated with 2 mL of distilled water each time.

(a) Group OS: OS files (25/6%) were used at the WL with a rotational speed of 400 rpm, and the torque was adjusted to 4 N.cm.
(b) Group WOG: The WOG Primary instrument (25/7%) was used in a reciprocating motion (using at 350 rpm with the “WAVEONE ALL” program). Slight apical pressure was applied during sample preparation. After short consecutive movements of penetration and removal, the instrument was removed from the canal and cleaned using a piece of sterile gauze. These procedures were repeated until the file reached the original WL, after which irrigation was performed following the same procedure as that described above.
(c) Group PTU: Initially, the SX file is utilized for cervical preparation. Following this, the S1 (17/2%), S2 (20/4%), F1 (20/7%), and F2 (25/8%) files are employed sequentially in a continuous clockwise motion at 300 rpm and 3 N.cm until reaching the WL.

### 2.4 Micro-CT analysis of tooth replicas

The resin replicas were scanned using micro-CT both before and after root canal instrumentation, following identical scanning and reconstruction protocols. No cases of instrument separation or strip perforation occurred during the procedures.

**Figure 2:**
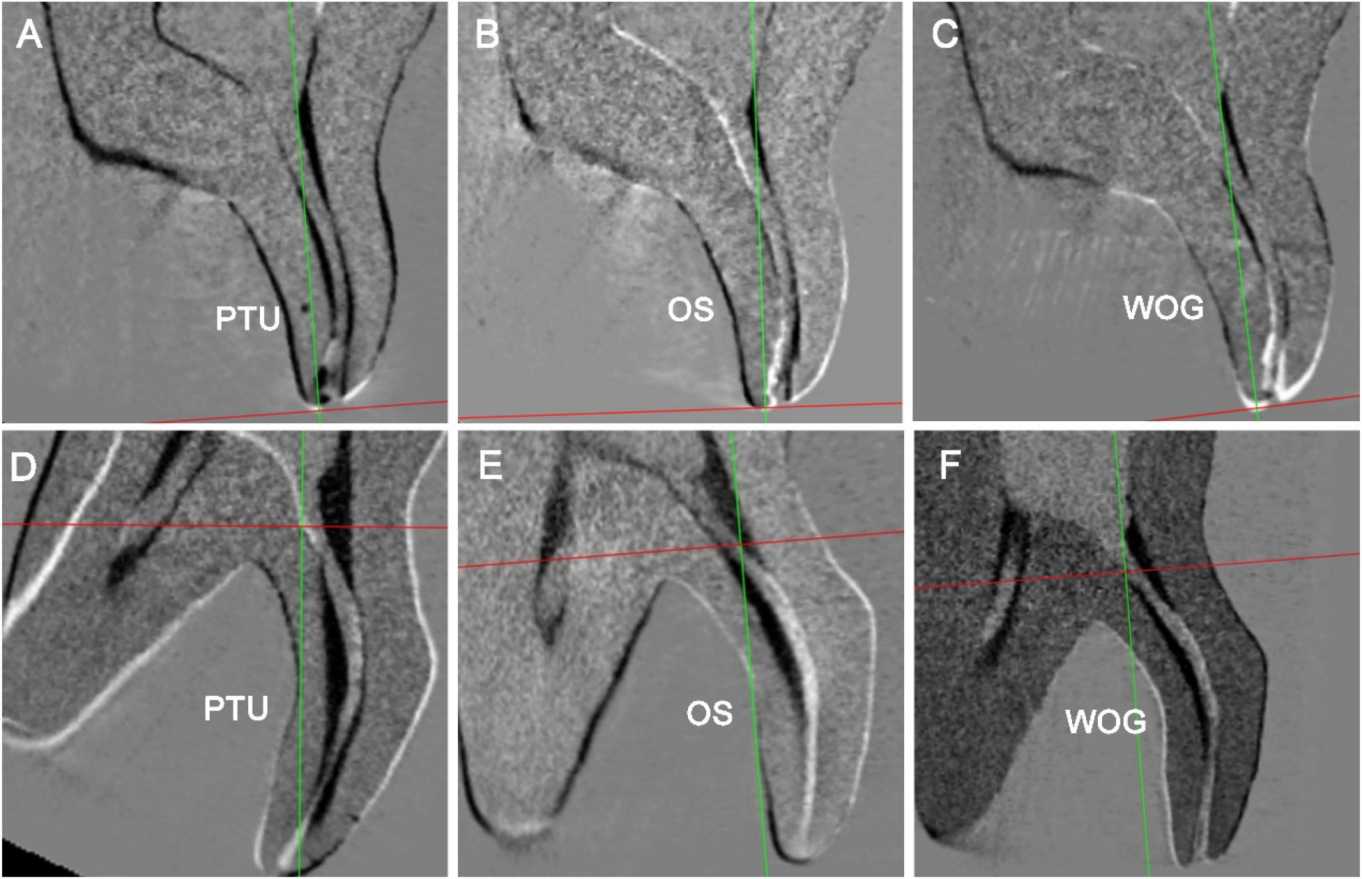
Representative micro-CT images of resin-printed three-rooted mandibular first molars (sagittal sections along the axes of the curved DL canal) showing pre- and post-instrumentation canal configurations of the DL canals after 3D registration. The black areas represent “dentin” removal zones. **(A–C)** Replicas from Specimen 1 prepared with PTU, OS and WOG respectively; **(D–F)** replicas from Specimen 2 prepared with PTU, OS and WOG respectively. Areas of resin removal are depicted in black.

**Figure 3:**
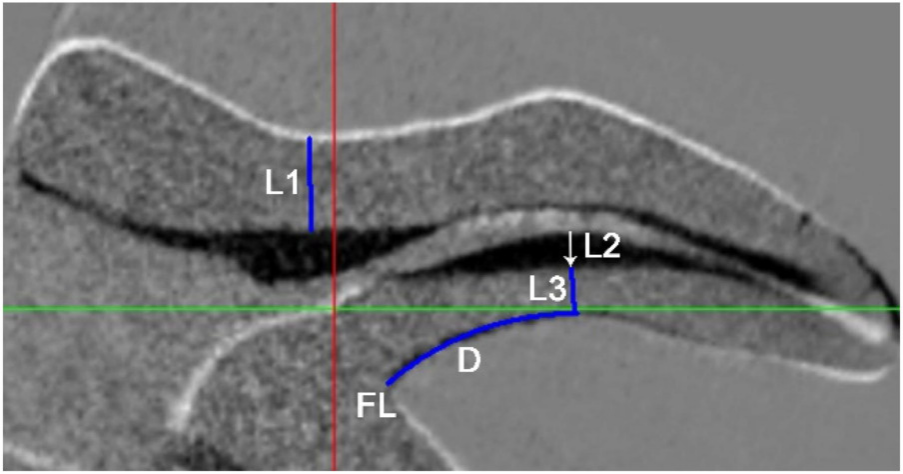
Measurement of the minimum remaining wall thickness (RWT) at the cervical (L1) and furcation (L3) regions, as well as the location **(D)** and magnitude (L2) of the maximum cutting thickness (MCT) after DL canal instrumentation (A representative micro-CT cross-sectional image obtained along the longitudinal axis of the DL canal before and after preparation, following 3D registration). Areas of resin removal are depicted in black.

Pre- and post-instrumentation images were aligned using the 3D registration module in Data Viewer software (v1.5.1, Bruker micro-CT, Belgium) (Figures 2, 3). Then, a section plane was initially set to approximate the sagittal plane to accommodate the characteristic buccolingual curvature of the DL canal, with subsequent adjustments made to optimally align with the canal's longitudinal axis and fully reveal its anatomical features. The selected sectional images were then exported in BMP format for quantitative analysis using Image-Pro Plus 6.0 (Media Cybernetics, USA). After calibration, the following measurements were performed:

Root canal straightening: The angles of root canal curvature were measured before and after instrumentation using Schneider's method (27) in the plane of curvature. Canal straightening was defined as the reduction in the angle of curvature following instrumentation.

**Figure 4:**
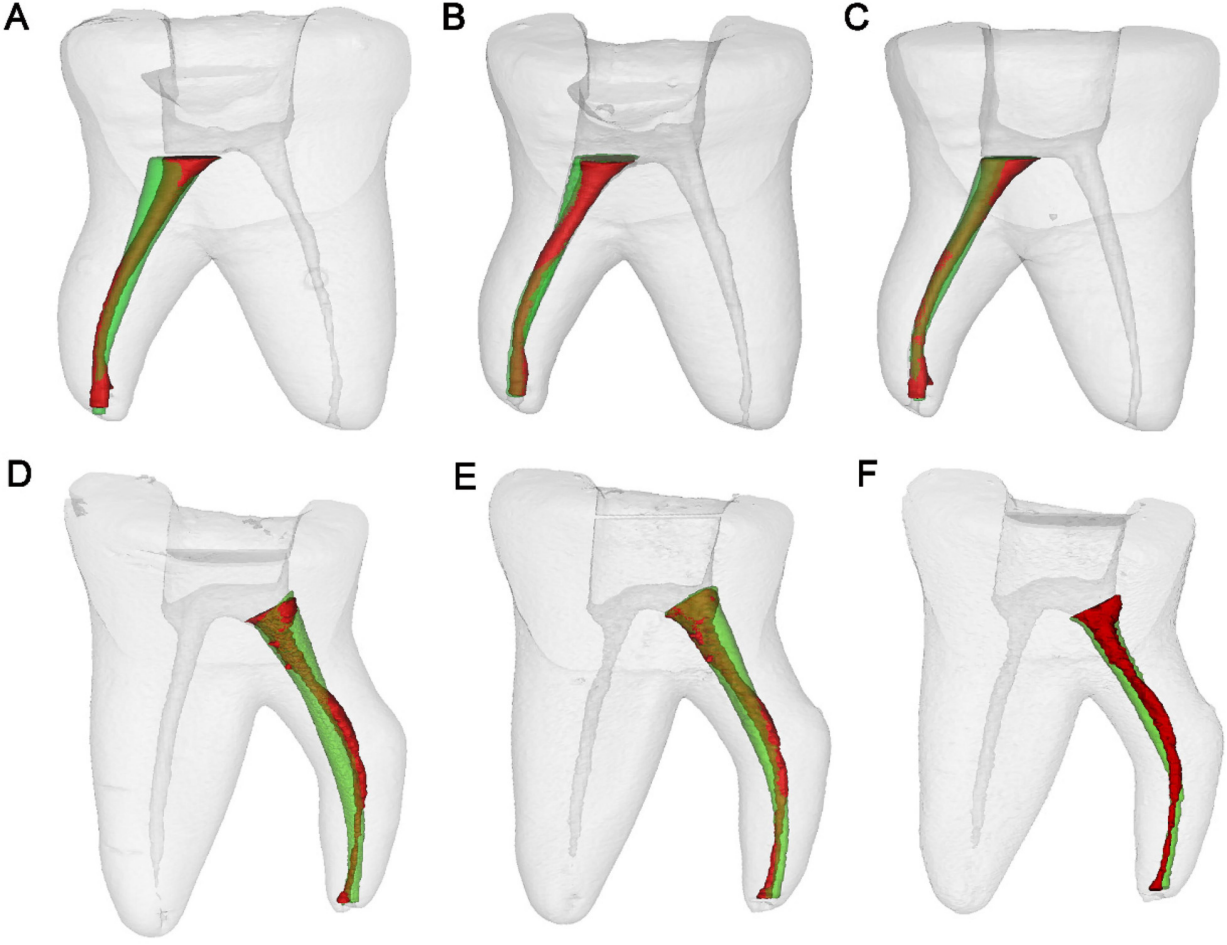
The superimposition of micro-CT 3D reconstruction models of the DL root canal before and after preparation (proximal view, red indicates before preparation, green indicates after preparation). **(A–C)** Specimen 1 replicas prepared with PTU, OS and WOG, respectively; **(D–F)** Specimen 2 replicas prepared with PTU, OS and WOG, respectively.

Canal volume and surface area: The 3D models of the teeth and root canal systems were reconstructed in Mimics software (Figure 4). Pre- and post-instrumentation volumes and surface areas of the DL canal were obtained. The percentage changes in volume/surface area (% Δ) was calculated using the formula: % Δ = ([A-B]/B) × 100, where A and B represent post- and pre-instrumentation data, respectively.

Minimum residual wall thickness (RWT) at the cervical region: Minimum RWT at the cervical region (L1) was measured at the lingual wall adjacent to the DL canal orifice (Figure 3).

Maximum cutting thickness (MCT) at the furcation region: The site and magnitude of MCT was identified along the inner (buccal) wall of the DL canal curvature. The MCT magnitude (L2) was defined as the greatest perpendicular distance between the pre- and post-instrumentation canal contours, representing the resin removal area (shown in black). The surface distance (D) from the furcation to the MCT site was measured along the DL root surface. To assess procedural safety, the residual buccal wall thickness (L3) at the MCT location was quantified (Figure 3).

### 2.5 Statistical analysis

Sample size calculation was performed using G*Power software (version 3.1.9.7; Heinrich-Heine Universität Düsseldorf, Düsseldorf, Germany). Based on pilot data and previous similar micro-CT studies evaluating canal volume changes and transportation during NiTi instrumentation, a medium-to-large effect size (Cohen's *f* = 0.60) was assumed for the primary outcome. With *α* = 0.05 and a desired power of 0.80, the analysis indicated a minimum total sample size of 31 specimens. Given the highly standardized nature of the 3D-printed replicas (which minimizes inter-specimen variability compared to extracted teeth), and common practices in comparable *ex vivo* endodontic studies (where group sizes typically range from 6 to 10) (24, 25), six replicas per group were selected (*n* = 6 per group; total *n* = 18 per prototype specimen, yielding 36 replicas overall). One-way analysis of variance (ANOVA) followed by Tukey's *post hoc* test was employed for multiple group comparisons. Student's *t*-test was conducted to compare the means between Specimen 1 (S1) and Specimen 2 (S2) replica groups. A significance level of 5% was used.

## 3 Results

### 3.1 The homogeneity of tooth replicas prior to instrumentation

Pre-instrumentation measurements revealed comparable morphometric parameters (surface area, volume, thickness, and curvature) of DL canals across all experiment groups (all *p* > 0.05), confirming the homogeneity of the tooth replicas prior to experimental interventions.

### 3.2 Canal straightening

Post-instrumentation analysis of canal straightening demonstrated statistically significant differences among three NiTi systems in both specimen groups (*p* < 0.01) (Table 2), with PTU causing the greatest straightening and WOG the least. Notably, Specimen 2 replicas (higher initial curvature) consistently showed more straightening than Specimen 1 replicas (*p* < 0.01).

**Table 2:** Measurement results of canal straightening of the DL canals (*n* = 6 per group).

NiTi systems	Apical size	Specimen 1 replicas	Specimen 2 replicas
OS	#25	3.9 ± 0.8^b^	9.0 ± 1.6^a^**
WOG	#25	2.5 ± 0.4^c^	4.8 ± 1.5^b^**
PTU	#25	5.4 ± 0.4^a^	11.0 ± 1.5^a^**
*F*		38.07	21.53
*P*		0.001	0.000

Values with the different lowercase superscript letters along the same column are significantly different (*p* < 0.05).

** Means statistically significant difference between replicas from Specimen 1 and Specimen 2 (Student's *t*-test, *p* < 0.01) (OS, OneShape; WOG, WaveOne Gold; PTU, ProTaper Universal).

### 3.3 Root canal volume and surface area

The measurement results of the pre- and post-instrumentation volume and surface areas of the DL canals were summarized in Table 3. WOG instrumentation demonstrated the minimal volumetric and surface area augmentation, contrasting with PTU which produced the most substantial expansion. Statistical significance emerged between PTU and WOG systems in both replica groups (*p* < 0.05), while OS exhibited intermediate dimensional changes.

**Table 3:** Measurement results of the initial and post-instrumentation volume and surface areas of the DL canals (*n* = 6 per group).

NiTi system	Initial Volume (mm^3^)	Post-instrumentation volume (mm^3^)	Volume increase (%)	Initial surface area (mm^2^)	Post-instrumentation surface area (mm^2^)	Surface area increased (%)
Specimen 1 replicas						
OS	2.14 ± 0.18	3.62 ± 0.57^ab^	57.9 ± 26.9^ab^	16.99 ± 0.91	22.44 ± 1.60^a^	28.4 ± 10.5^ab^
WOG	2.29 ± 0.16	2.86 ± 0.21^b^	30.8 ± 13.1^b^	17.50 ± 0.79	18.61 ± 0.76^b^	10.1 ± 8.8^b^
PTU	2.19 ± 0.16	4.37 ± 0.58^a^	106.9 ± 41.0^a^	18.35 ± 0.94	23.74 ± 2.21^a^	40.3 ± 17.5^a^
*F*	1.118	12.14	8.67	0.599	13.23	7.207
*P*	0.359	0.001	0.005	0.565	0.001	0.009
Specimen 2 replicas						
OS	2.04 ± 0.14	4.39 ± 1.10^ab^	118.4 ± 67.1^ab^	17.86 ± 1.36	23.56 ± 3.14^ab^	31.8 ± 12.7^ab^
WOG	2.11 ± 0.13	3.79 ± 0.88^b^	78.6 ± 35.3^b^	17.82 ± 0.94	21.97 ± 2.36^b^	23.6 ± 15.3^b^
PTU	2.13 ± 0.14	5.84 ± 0.51^a^	174.6 ± 26.8^a^	17.96 ± 0.91	28.64 ± 1.75^a^	60.0 ± 16.7^a^
*F*	1.040	8.540	7.28	0.155	8.960	8.180
*P*	0.594	0.014	0.026	0.925	0.011	0.017

Values with the different lowercase superscript letters along the same column are significantly different (*p* < 0.05). (OS, OneShape; WOG, WaveOne Gold; PTU, ProTaper Universal).

### 3.4 MCT and residual wall thickness at the cervical and furcation regions

The measurement results for the MCT and minimum RWT at the cervical and furcation regions post-instrumentation are presented in Table 4. The WOG system demonstrated superior structural conservation, exhibiting the smallest MCT, whereas the PTU system showed the greatest MCT. Furthermore, assessment of structural integrity indicated that WOG preserved more tooth structure in both cervical and furcal areas, with significantly larger RWT values across all tooth replica sets (both *p* < 0.01). The MCT location—a critical perforation risk indicator—was observed at a significantly greater mean surface distance below the furcation in PTU specimens (3.0 ± 0.3 mm for S1; 2.5 ± 0.4 mm for S2) compared to WOG specimens (2.2 ± 0.2 mm for S1; 2.1 ± 0.3 mm for S2), whereas the OS system showed intermediate values (2.2 ± 0.3 mm for S1; 2.4 ± 0.2 mm for S2).

**Table 4:** Measurement of remaining wall thickness (RWT), and canal transportation after DL canal instrumentation (mm, *n* = 6 per group).

NiTi System	Minimum RWT at cervical region (L1)	RWT at MCT level (L3)	Level of MCT (D)	MCT (L2)
Specimen 1 replicas				
OS	1.50 ± 0.06^b^	1.29 ± 0.02^a^	2.37 ± 0.24^a^	0.24 ± 0.03^ab^
WOG	1.57 ± 0.03^b^	1.38 ± 0.03^b^	2.05 ± 0.09^b^	0.20 ± 0.04^b^
PTU	1.37 ± 0.06^a^	1.28 ± 0.02^a^	2.54 ± 0.17^a^	0.28 ± 0.04^a^
*F*	19.16	10.01	11.92	11.92
*p*	0.000	0.002	0.001	0.001
Specimen 2 replicas				
OS	1.46 ± 0.09^a^	0.89 ± 0.13^ab^	2.24 ± 0.14^b^	0.34 ± 0.05^ab^
WOG	1.63 ± 0.12^b^	0.91 ± 0.02^b^	2.19 ± 0.62^b^	0.30 ± 0.07^b^
PTU	1.32 ± 0.06^a^	0.73 ± 0.06^a^	2.99 ± 0.32^a^	0.39 ± 0.04^a^
*F*	13.650	6.713	6.011	4.143
*p*	0.001	0.011	0.016	0.037

Values with the different lowercase superscript letters along the same column are significantly different (*p* < 0.05). (OS, OneShape; WOG, WaveOne Gold; PTU, ProTaper Universal).

RWT denotes remaining wall thickness, and MCT denotes maximum canal transportation.

## 4 Discussion

In this investigation, two permanent mandibular first molars with DL roots, representing varying degrees of curvature, were selected from a collected sample pool. Corresponding resin replicas were then fabricated using 3D printing technology. Pre-operative geometric measurements indicated no significant differences among three experimental groups (all *p* > 0.05) across each set of tooth replicas, indicating that the replicas possessed a consistent canal morphology. Traditional *ex vivo* studies evaluating root canal instrumentation have predominantly utilized extracted human teeth. However, the inherent anatomical variability of natural teeth poses significant challenges for the establishment of balanced experimental groups. Furthermore, the DL root typically exhibits severe curvature and reduced dimensions, making it particularly susceptible to fracture during the extraction process. Consequently, procuring sufficient quantities of intact DL root specimens for instrumentation studies remains problematic. Our results corroborate previous studies (22–25), demonstrating that 3D-printed tooth replicas offer advantages in terms of consistency, reproducibility, and repeatability, making them an excellent model for assessing the performance of various instruments. These replicas effectively simulate the clinical conditions and challenges involved in preparing a DL canal in 3RM1s.

The DL root typically presents with a narrow, conical canal and a pronounced buccal curvature (2, 7). This complex anatomy increases the risk of procedural errors during instrumentation. Recent advancements in NiTi file systems—particularly single-file systems—have revolutionized root canal preparation by combining flexibility and strength to optimize shaping in curved canals (12–14). These innovations address two key objectives: (1) preservation of the original canal anatomy and (2) efficient dentin removal, thereby reducing procedural time and minimizing instrument fatigue. Moreover, enhancements in alloy composition and manufacturing techniques have significantly reduced the incidence of unexpected NiTi instrument separation, a historically prevalent concern. Contemporary NiTi systems such as OS, WOG, and PTU have demonstrated satisfactory efficacy in preparing curved canals (12–16, 27). However, their geometric designs, alloy enhancements, and kinematic properties vary significantly. To date, no studies have specifically evaluated their performance in addressing the unique challenges posed by DL canal anatomy.

The present study found no occurrences of instrument separation or strip perforation across all experimental groups. However, the significantly higher incidence of procedural errors associated with the PTU system indicates that the three NiTi systems cannot be regarded as equally safe for DL canal preparation. It is well established that excessive use of rigid files in curved canals may lead to undesirable canal straightening. Instrument flexibility is determined by multiple factors such as instrument geometry, alloy composition, thermomechanical processing, and surface treatments (9). Comparative analysis revealed that the WOG system produced minimal canal straightening (Table 2). This superior performance can be attributed to its unique design features: an off-center parallelogram cross-section with dual cutting edges, a reduced 7% taper (primary), and a specialized reciprocating motion that reduces torsional stress. Additionally, the manufacturing process incorporates gold heat treatment—a precisely controlled thermal protocol involving gradual heating and cooling cycles—which substantially improves the instrument's mechanical properties. These synergistic design innovations collectively enable superior maintenance of original canal curvature during instrumentation (14–17). Conversely, the PTU system employs a progressively increasing taper design, with the final instrument (F2) reaching an 8% taper (18, 19). When combined with its conventional NiTi alloy composition and larger cross-sectional core area (featuring a convex triangular configuration), this system exhibits markedly increased rigidity. Additionally, the extended instrumentation duration required by PTU's multi-file protocol resulted in significantly greater canal straightening compared to the single-file systems. The OS system, as a first-generation single-file instrument, maintains a constant 6% taper and incorporates an innovative cross-sectional design (a triangle-shaped symmetrical three-cutting-edge in the tip area, a S-shaped symmetrical two-cutting-edge at the coronal area, and an asymmetrical progressively changing in the middle) (12, 28, 29). While its reduced core diameter and optimized geometry provide enhanced flexibility—resulting in less canal deviation than PTU—its conventional austenite alloy composition limited its performance relative to the advanced WOG system.

Table 3 reveals that instrumentation with the PTU system produced significantly greater resin removal compared to the single-file systems, creating the largest post-instrumentation canal dimensions. This outcome may be attributed to PTU's progressively increasing taper design and the crown-down preparation. While coronal flaring facilitates apical access by eliminating cervical constrictions and reduces taper lock and frictional forces (30), it inevitably alters native canal morphology, particularly in cervical regions. Such morphological changes warrant careful consideration, especially when addressing the challenging anatomy of DL canals characterized by their narrow dimensions and complex curvature. In contrast, the OS and WOG systems employ conservative taper designs (6% and 7%, respectively) without requiring coronal flaring. When maintaining identical apical preparation size (25#), the longitudinal cross-sectional area directly correlates with taper size, explaining why PTU's 25/8% (F2) instrument produced the greatest canal enlargement. The single-file systems demonstrated several clinical advantages, including reduced preparation time, enhanced flexibility, and maintained structural strength. Notably, WOG's gold heat-treated alloy exhibits superior superelastic properties that reduces cutting forces and frictional resistance while improving curvature negotiation (14–17), resulting in less aggressive dentin cutting, prevention of procedural errors, and consequently the least resin removal among all tested systems.

The comparative analysis of canal curvature cross-sections revealed significant variations among the instrumentation systems, as quantitatively demonstrated in Table 4 and visually illustrated in Figures 2, 4. Notably, the WOG system maintained optimal anatomical conservation with minimal deviation from the original canal path and least MCT at the furcation region. In contrast, the PTU group exhibited the most pronounced structural alterations. Analysis of resin removal patterns identified three primary sites of predilection: (1) the lingual aspect of the DL canal orifice, (2) the inner (buccal) wall of the curvature at the furcation region, and (3) the outer (lingual) wall of the apical canal curvature. This characteristic spatial distribution revealed the non-uniform resin/dentin removal during instrumentation, with PTU specimens exhibiting the most significant procedural errors. Previous studies have demonstrated that excessive dentin removal during root canal preparation elevates the risk of root fracture (31, 32). The DL root's pronounced buccolingual curvature and narrow dimensions increase its susceptibility to iatrogenic complications such as strip perforation and instrument fracture during endodontic procedures. Table 3 reveals that the location of MCT—a critical indicator of perforation risk—was consistently located 2–3 mm below the furcation. This region frequently corresponds to areas demonstrating both the most extensive resin removal and the thinnest RWT. While this location parallels the classical “danger zone” observed in mesial roots of mandibular molars (33), the DL root exhibits greater anatomical variability (1, 2, 34). Moreover, the morphology of the distal furcation between the two distal roots is another influence factor (7). A short root trunk combined with a pronounced divergence angle between the DB and DL roots confers a predisposition to perforation.

Recent biomechanical evidence demonstrated that the pericervical region is particularly susceptible to fracture under functional and parafunctional occlusal forces (35). Our findings indicate that this vulnerability is exacerbated by the PTU system, which utilizes an aggressive crown-down approach and large-taper design that remove excessive cervical dentin/resin. In contrast, the WOG system, representing the latest generation of single-file instrumentation, demonstrates superior flexibility. Its gold heat treatment process optimizes cutting efficiency while reducing the risk of over-preparation. Furthermore, WOG's reciprocating motion—engaging dentin counterclockwise before disengaging clockwise—minimizes torsional stress and prevents taper lock, thereby enhancing safety. These attributes allow WOG to achieve an optimal balance between efficacy and preservation, making it particularly suitable for DL canal preparation. While the OS system demonstrates intermediate performance between WOG and PTU, its smaller taper (6%) and reduced cross-sectional core area improve flexibility. However, as a first-generation single-file system made from conventional NiTi alloy, OS exhibits inferior shaping ability compared to WOG. Moreover, OS operates with continuous 360 ° rotation at 400 rpm, providing greater cutting efficiency, which may lead to more aggressive resin removal than the reciprocating system. Therefore, in cases of pronounced DL root curvature (e.g., Specimen 2), meticulous technique remains essential to minimize dentin/resin removal in the cervical and furcation regions. Overall, the significant differences observed in all evaluated parameters led to rejection of the null hypothesis. The single-file systems (WOG and OS) demonstrated superior shaping ability compared to the multi-file PTU system in the DL canals.

Clinically, these findings provide valuable guidance for managing 3RM1s—an anatomical variant with marked ethnic predisposition and a frequent source of diagnostic oversight or iatrogenic complications (2, 6). The superior conservation of canal anatomy and reduced risk of excessive dentin removal achieved with WOG, especially in severely curved DL canals, translates to a lower risk of procedural errors such as furcation strip perforation and excessive pericervical dentin removal, both of which are established predictors of vertical root fracture and compromised long-term tooth survival (31, 32, 35). By minimizing structural weakening, single-file systems like WOG and OS may enhance post-treatment prognosis and reduce the need for subsequent restorative or surgical interventions. Additionally, their streamlined workflow—requiring fewer instruments—offers practical benefits in clinical practice, including reduced preparation time and lower risk of cross-contamination due to single-use protocols, while maintaining comparable or superior shaping outcomes compared with multi-file systems (12, 25). In contrast, the more aggressive material removal associated with PTU underscores the potential drawbacks of traditional progressive-taper approaches in narrow, highly curved canals, where over-preparation could precipitate early tooth loss despite successful obturation. These results reinforce the need for individualized instrument selection informed by preoperative imaging (e.g., CBCT) to identify complex anatomies, particularly in populations with elevated prevalence of radix entomolaris. Ultimately, the adoption of advanced heat-treated reciprocating single-file systems (e.g., WOG) may contribute to higher treatment success rates and improved patient-centered outcomes in challenging endodontic scenarios, warranting further validation through randomized clinical trials.

This study has several limitations. First, the 3D-printed 3RM1s were based on replicas of only two human specimens. Although these models represent different curvature severities, their restricted sample size may introduce selection bias regarding other anatomical parameters. Second, the root curvatures were approximately confined to the buccolingual plane, whereas natural teeth often exhibit complex 3D curvatures, potentially underestimating the procedural challenges encountered in clinical practice. Third, although 3D-printed replicas offer substantial advantages over extracted teeth, including precise control of anatomical parameters, perfect standardization across experimental groups, elimination of inter-specimen variability, avoidance of ethical and logistical issues associated with tooth collection and storage, reduced risk of cross-contamination, and the ability to produce unlimited identical replicas for repeatable testing, these models remain an *ex vivo* simulation. Finally, the rigid photopolymer resin used has a Shore D hardness of ∼84, comparable to cured PMMA (typical range for acrylic denture teeth: 80–90), and may not fully replicate the mechanical properties of natural dentin (e.g., elasticity, fracture toughness, or tactile feedback during instrumentation), potentially influencing instrumentation outcomes and procedural errors. Therefore, although 3D-printed replicas effectively overcome key limitations of extracted teeth, particularly the challenges in obtaining sufficient intact DL roots due to their fragility and high anatomical variability, the findings from this standardized model should be interpreted cautiously and validated with natural teeth or *in vivo* studies before clinical extrapolation.

## 5 Conclusions

In conclusion, both single-file systems (WOG and OS) demonstrated satisfactory performance in shaping DL canals of 3RM1s. In contrast, the PTU system consistently exhibited excessive and asymmetrical resin/dentin removal, potentially compromising root structural integrity and increasing the likelihood of iatrogenic complications. Consequently, PTU appears less suitable for DL canal preparation compared to the more conservative single-file systems.

## Data Availability

The original contributions presented in the study are included in the article/Supplementary Material, further inquiries can be directed to the corresponding author.
